# Cerulean cataract mapped to 12q13 and associated with a novel initiation codon mutation in MIP

**Published:** 2011-07-26

**Authors:** Xueshan Xiao, Wei Li, Panfeng Wang, Lin Li, Shiqiang Li, Xiaoyun Jia, Wenmin Sun, Xiangming Guo, Qingjiong Zhang

**Affiliations:** 1 State Key Laboratory of Ophthalmology, Zhongshan Ophthalmic Center, Sun Yat-sen University, Guangzhou, China; 2 Department of Ophthalmology, Shenzhen Second Hospital, Shenzhen, China

## Abstract

**Purpose:**

To identify the genetic defect in a large Chinese family with autosomal dominant cerulean cataract.

**Methods:**

Genomic DNA and clinical data were collected from the family. Candidate gene sequencing and genome-wide linkage analysis were used to disclose the molecular basis responsible for cerulean cataract in the family.

**Results:**

Initially, sequencing analysis of the three genes (beta-B2-crystallin [*CRYBB2*], gamma-D-crystallin [*CRYGD*], and V-MAF avian musculoaponeurotic fibrosarcoma oncogene homolog [*MAF*]) known to cause cerulean cataract failed to find any mutation. Then, genome-wide linkage analysis mapped the disease to chromosome 12q13-q22 between D12S85 and D12S351, with a maximum lod score of 4.10 at θ=0. Sequence analysis of the major intrinsic protein of lens fiber gene (*MIP*), a gene known to cause other types of cataract in the linkage interval, detected a novel heterozygous initiation codon mutation, c.2T>C (p.Met1?). This mutation was present in all patients with cerulean cataract but was not present in any of the 13 unaffected family members nor in 96 control individuals.

**Conclusions:**

Cerulean cataract was found in a large family and is caused by a novel initiation codon mutation in *MIP*. This study adds a new member in the existing list of genes causing cerulean cataract and expands the mutation spectrum and phenotypic association of *MIP* mutations.

## Introduction

About one third of infant blindness is due to congenital cataracts [1,2]. Congenital cataracts can result in significant vision loss by impairing visual development. Genetic factors played an important role in the development of congenital cataracts, with up to 25% of them hereditary [3-5]. Hereditary congenital cataracts can be inherited as an autosomal dominant, autosomal recessive, or X-linked trait, where the autosomal dominant trait is most commonly described. So far, mutations in at least 21 genes have been identified to be responsible for a subset of nonsyndromic congenital cataracts while a substantial number of causative genes remain to be determined [6,7].

Clinical phenotypes of hereditary congenital cataract are highly heterogeneous. Specific clinical signs may be more frequently related to one or a few causative genes but establishment of genotype-phenotype correlation is usually difficult in most cases. Cerulean cataract (OMIM 115660) is a specific type of cataract characterized by predominantly bluish opacifications in the superficial layers of the fetal nucleus as well as the adult nucleus of the lens. At least four loci for cerulean cataract have been identified, including cerulean type congenital cataract-1 (CCA1; OMIM 115660,17q24) [8], CCA2 (OMIM 601547, 22q11.2-q12.2) [9], CCA3 (OMIM 608983, 2q33-q35) [10], and CCA4 (OMIM 610202, 16q22-q23) [11]. Mutations in 3 genes have been identified to be responsible for cerulean cataract, i.e., the beta-B2-crystallin gene (*CRYBB2*, OMIM 123620) [12], the gamma-D-crystallin gene (*CRYGD*, OMIM 123690) [12], and the V-MAF avian musculoaponeurotic fibrosarcoma oncogene homolog gene (*MAF*, OMIM 177075) [11].

In this study, cerulean cataract was found in a five-generation Chinese family. An initial scan of the three genes known to cause cerulean cataract did not detect any mutation. A subsequent genome-wide linkage study mapped the cerulean cataract locus to chromosome 12q13-q22. Sequencing the candidate gene in the linkage interval identified a novel c.2T>C (p.Met1?) mutation in the major intrinsic protein of lens fiber gene (*MIP*, OMIM 154050).

## Methods

### Family with cerulean cataract

A five generation family with congenital cerulean cataract was identified from the Eye Hospital of Zhongshan Ophthalmic Center, Sun Yat-sen University, Guangzhou, China. The family was originated from Central China and moved to Shenzhen, China in recent years. Written informed consent was obtained from the participating individuals or their guardians before the collection of clinical data and genomic samples. This study was approved by the Internal Review Board of the Zhongshan Ophthalmic Center and followed the tenets of the Declaration of Helsinki and the Guidance of Sample Collection of Human Genetic Diseases (863-Plan) by the Ministry of Public Health of China.

Genomic DNA was prepared from venous leukocytes [13].

### Mutational screening

Bioinformation of *CRYBB2*, *CRYGD*, and *MAF* was obtained from the National Center for Biotechnology Information (NCBI). Polymerase chain reaction (PCR) was used to amplify the coding exons and adjacent intronic sequences of the 3 genes using the primers referred to the previous publication [14] with modification (Table 1). PCR amplifications were carried out in 20-μl reactions containing 80 ng genomic DNA. PCR cycles consisted of a denaturizing step at 95 °C for 5 min, followed by 35 cycles of amplification (at 95 °C for 30 s, at 53.5~69°C for 30~60 s for 35~40 cycles, and at 72 °C for 30 s), and a final extension at 72 °C for 5 min. The nucleotide sequences of PCR products were determined with the ABI BigDye Terminator cycle sequencing kit v3.1 on an ABI 3100 genetic analyzer (ABI Applied Biosystems, Foster City, CA). Variations were identified by importing the sequencing results from patients and consensus sequences from the NCBI human genome database into the SeqManII program of the Lasergene package (DNAStar Inc., Madison, WI).

**Table 1:** Primers used to amplify genomic fragments of candidate genes.

**Gene name**	**Primer ID**	**Primer sequence (5'-3')**	**Product length (bp)**	**Annealing temperature (°C)**
*CRYBB2*	1F	TTGGGGCCAGAGGGGAGTG		
* *	1R	TGGGCTGGGGAGGGACTTTC	353	66
* *	2F	AGGTCCCACGGCTGCTTAT		
* *	2R	GGCTGCCAGACCCCAAAACT	421	64
* *	3F	GTGGGTAAAGGCAGCATAGC		
* *	3R	GGCAGAGAGAGGGAGTAGGG	378	68
* *	4F	GGTGCACTGGGAAGAGAGTG		
* *	4R	GAAGCCAGAGGTCAGCAGAG	397	60
* *	5F	GAGGCTTCACCCTTCCTAGTG		
* *	5R	GCAGACAAGTTGCAAGTCAC	389	69
*CRYGD*	1-2F	GGGCCCCTTTTGTGCGGTTCT		
* *	1-2R	GTGGGGAGCAAACTCTATTGA	643	65
* *	3F	TGCTCGGTAATGAGGAGTTT		
* *	3R	AAATCAGTGCCAGGAACACA	506	63
*MAF*	1aF	GAGCGAGGGAGCACATTG		
* *	1aR	CCGGTTCCTTTTTCACTTCA	352	60
* *	1bF	AACTGGCAATGAGCAACTCC		
* *	1bR	GTGGTGGTGGTGGTGGTAGT	548	60
* *	1cF	CCGCACTACCACCACCAC		
* *	1cR	CTGGTTCTTCTCCGACTCCA	432	60
* *	1dF	AGCTGGTGACCATGTCTGTG		
* *	1dR	AGAACTAGCAAGCCCACACC	407	53.5
* *	2F	AAATCCTGAGTAAGTGCCATTCA		
* *	2R	GTTGCATTCCGGGAAACTT	575	60
*MIP*	1F	GACTGTCCACCCAGACAAGG		
	1R	TCAGGGAGTCAGGGCAATAG	492	64~57
	2F	TGAAGGAGCACTGTTAGGAGATG		
	2R	AGAGGGATAGGGCAGAGTTGATT	500	64~57
	3F	CCAGACAGGGCATCAGT		
	3R	TGGTACAGCAGCCAACAC	373	64~57
	4F	AAGGTGTGGGATAAAGGAGT		
	4R	TTCTTCATCTAGGGGCTGGC	429	64~57
	SeqE1R	AAGGCACGGAGCAGGGACATC		

### Genotyping and linkage analysis

Genotyping for all participating family members was performed using 5′-fluorescently labeled microsatellite markers as previous described [15]. Briefly, a genome-wide scan was carried out using panels 1 to 27 of the ABI PRISM linkage Mapping Set Version 2 (Applied Biosystems). PCR was conducted at 94 °C for 8 min, followed by 10 cycles of amplification at 94 °C 15 s, 55 °C 15 s, and 72 °C 30 s; then 20 cycles at 89 °C 15 s, 55 °C 15 s, 72 °C 30 s; finally at 72 °C for 10 min. After mixing with GENESCAN^™^ 400HD (ROX^™^) standard (Applied Biosystems) and deionized formamide, the amplicons were denatured at 95 °C for 5 min and then immediately placed on ice for 5 min. The amplicons were separated on an ABI 3100 Genetic Analyzer (Applied Biosystems). Genotyping data were analyzed using the Gene Mapper version 3.5 software package (Applied Biosystems). Two-point linkage analysis was performed by using the MLINK program of the FASTLINK implementation of the LINKAGE program package [16,17]. The cerulean cataract in the family was analyzed as an autosomal dominant trait with full penetrance and with a disease-gene allele frequency of 0.0001. Haplotypes were generated using the Cyrillic 2.1 program (Cyrillic Software, Wallingford, Oxfordshire, UK) and confirmed by inspection. The criteria for establishing linkage have been previously described. Briefly, a lod score of 3 is accepted as significant evidence for linkage for autosomal diseases while a lod score of 2 is considered to be significant linkage for X-linked diseases [18,19].

### Mutation identification in *MIP*

Primers used to amplify the 4 coding exons and their adjacent intronic region of MIP were the same as those in the previous report [20], except that a new primer was synthesized for additional reverse sequencing of exon 1 (Table 1). PCR amplifications were performed in 20-μl reactions containing 80 ng genomic DNA. Touchdown PCR amplification consisted of a denaturizing step at 95 °C for 5 min, followed by 35 cycles of amplification (at 95 °C for 30 s, at 64~57 °C for 30 s starting from 64 °C with decreasing by 0.5 °C every cycle for 14 cycles until remaining at 57 °C for 21 cycles, and at 72 °C for 40 s), and a final extension at 72 °C for 10 min. The nucleotide sequences of PCR products were determined with an ABI BigDye Terminator cycle sequencing kit v3.1 as described in the section for mutational screening. Any variant detected was initially confirmed by bidirectional sequencing and then evaluated in 192 chromosomes of 96 normal controls. Mutation description followed the recommendation of the Human Genomic Variation Society (HGVS).

## Results

**Figure 1:**
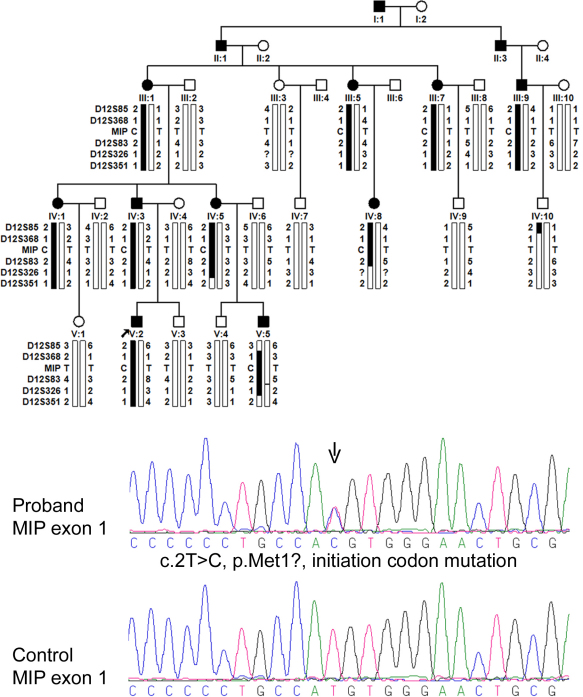
Pedigree, haplotypes on chromosome 12q and *MIP* mutation. Pedigree and haplotypes are shown on top. Filled squares (male) or circles (female) represent individuals affected with cerulean cataract. Bars filled with black indicate the chromosomal regions that are derived from the ancestral disease-associated haplotype. Sequence tracing of the *MIP* mutation is shown at bottom. Arrow indicates the site with double peaks, where a heterozygous T to C variant affects the second nucleotide of the ATG initiation codon for MIP.

**Figure 2:**
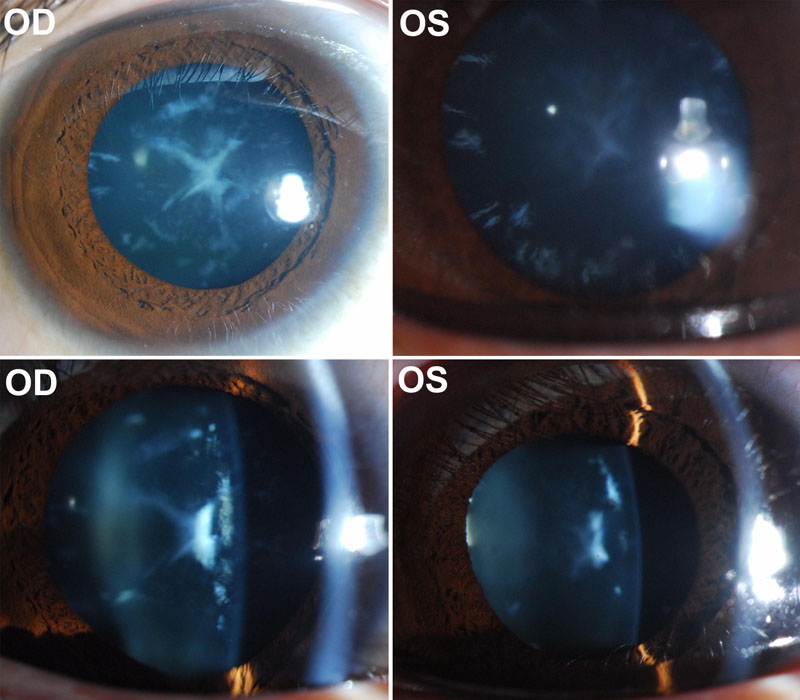
Lens photograph of the proband (V2). The photos were taken when the proband (V:2) was 19 years old. Cerulean cataract was observed in the right (OD) and left (OS) eyes, showing lamellar, Y-suture, and punctate lens opacities.

The disease in the family passed at least five generations. Twenty three individuals, including 10 affected and 13 unaffected, participated in this study (Figure 1). The cataract in all subjects were cerulean (Figure 2), but with different morphology (lamellar, punctuate, and/or Y-sutural) in different patients (Table 2). Although affected subjects complained of visual blur, their visual acuity is within the normal range or only mildly reduced. The fundus was normal in 17 eyes of the 10 affected subjects. Macular degeneration was observed in two eyes of one affected subject and traumatic retinal detachment was present in one eye of one affected subject. Nine affected subjects tested had normal color vision. Systemic examination did not find any significant abnormality.

**Table 2:** Clinical information of the affected family members in the family.

** **	** **	** **	**Visual acuity**		** **	** **	** **
**ID #**	**Gender**	**Age**	**OD**	**OS**	Cataract phenotype*	**Fundus OU (OD;OS)**	**Color vision**
III:1	F	66	0.04	0.1	C, L, Y	MD	N/A
III:5	F	52	1.0	1.0	C, L, P	normal	normal
III:7	F	45	0.8	0.5	C, L, P	normal	normal
III:9	M	44	1.0	1.0	C, L, P, Y	normal	normal
IV:1	F	45	1.0	1.0	C, L, P	normal	normal
IV:3	M	42	HM	1.2	C, L, P, Y	TRD;normal	normal
IV:5	F	39	1.0	1.0	C, L, P, Y	normal	normal
IV:8	F	24	1.2	1.2	C, L, P	normal	normal
V:2	M	19	0.8	1.2	C, L, P, Y	normal	normal
V:5	M	8	1.0	1.0	C, P	normal	normal

*Note: C=cerulean; L=lamellar; p=punctate; Y=Y suture. MD=macular degeneration; TRD=traumatic retinal detachment.

Initially, three genes known to cause cerulean cataract including *CRYBB2*, *CRYGD*, and *MAF* were analyzed by Sanger dideoxy sequencing. After complete analysis of the coding and adjacent intronic regions of the three genes, no mutation was identified. Then, a genome-wide linkage analysis was performed. Genome wide linkage scan mapped the cerulean cataract locus to chromosome 12q13-q22 between D12S85 and D12S351, with a maximum lod score of 4.10 at θ=0 (Figure 1, Table 3). One gene known to cause other types of cataract is present in the linkage interval, i.e., *MIP*. Subsequently, sequencing the coding regions of *MIP* identified a novel heterozygous c.2T>C (p.Met1?) mutation in exon 1 (Figure 1). The heterozygous c.2T>C mutation was present in all subjects with cerulean cataract but neither in any of the 13 unaffected family members nor in 96 control individuals. The mutation itself could establish linkage with a maximum lod score of 3.8 at θ=0 (Table 3). The c.2T>C mutation affect the initiation codon, which may result in no production of protein or activation of a new translation initiation site.

**Table 3:** Two-point lod scores of family for markers around *MIP*.

** **	**Position**		**Lod score at θ=**						
**Markers**	cM*	**Mb#**	**0.00**	**0.01**	**0.05**	**0.10**	**0.20**	**0.30**	**0.40**
D12S85	62.70	47.34	-inf	-0.53	0.69	1.04	1.07	0.79	0.39
D12S368	67.30	52.63	1.62	1.62	1.58	1.47	1.16	0.79	0.41
*MIP*	** **	56.85	3.80	3.73	3.43	3.05	2.24	1.40	0.59
D12S83	76.50	60.89	4.10	4.03	3.73	3.34	2.52	1.63	0.73
D12S326	87.60	77.97	1.80	1.76	1.59	1.38	0.94	0.51	0.16
D12S351	97.10	91.91	-inf	-0.07	1.07	1.33	1.24	0.88	0.43

*Genethon. #Homo genome (Build 37.2) Chr1 Primary_Assembly.

## Discussion

Previously, mutations in *CRYBB2*, *CRYGD*, and *MAF* have been identified to be responsible for three types of cerulean cataract [9-12], i.e., CCA2, CCA3, and CCA4. The causative gene for CCA1 at 17q24 is still unknown [8]. Here in this study, we identified a large family with autosomal dominant cerulean cataract. Initial mutational screening excluded *CRYBB2*, *CRYGD*, and *MAF* as the causative genes. A genome wide linkage analysis mapped the disease to chromosome 12q13-q22. Sequence analysis of *MIP* in the linkage interval identified a novel heterozygous c.2T>C mutation that cosegregated with the cataract and was not present in controls. All lines of evidence suggest that the *MIP* mutation is the cause of cerulean cataract in this family. This might add a new member in the existing list of genes causing cerulean cataract when mutated.

Nine different mutations in *MIP* have been identified in 9 families with different types of autosomal dominant cataract, including seven missense mutations (c.97C>T [p.R33C] [21], c.319G>A [p.V107I] [22], c.401A>G [p.E134G] [23], c.413C>G [p.T138R] [23], c.530A>G [p.Y177C] [24], c.559C>T [p.R187C] [25], and c.698G>A [p.R233K] [26]), one splicing site mutation that activates a cryptic splicing acceptor in the 3′UTR region (c.607–1G>A [p.V203fs] [20]), and one deletion resulted in framshift (c.638delG [p.G213VfsX46] [27]). These mutations are located in exon 1 (2 families), exon 2 (2 families), exon 3 (2 families), and exon 4 (3 families, including 1 families in 3′ end of intron 3). Initiation codon mutation identified in this study represents a different type of mutation, which has been rarely reported in cataract. Of the great number of mutations identified in nearly 200 genes and loci [7], only two mutations involving the initiation codon have been reported in beta B1-crystallin (*CRYBB1*) and galactokinase 1 (*GALK1*), respectively [28,29].

Previously, phenotypes of the cataract in the 9 families with *MIP* mutation included nuclear polymorphic and lamellar [23]; punctuate and lamellar [30]; nuclear punctuate, suture, and cortical [27]; total [21]; punctate and polymorphic [26]; snail-like [20]; Y-sutural, nuclear pulverulent, and nuclear [22]; Nuclear [25]; congenital nuclear [24]. Cerulean cataract as a major finding has not been described in the previous studies.

In summary, cerulean cataract was found in a large family and is caused by a novel initiation codon mutation in *MIP*. This study expands the mutation spectrum and phenotypic association of *MIP* mutations.

## References

[1] Robinson GC, Jan JE, Kinnis C (1987). Congenital ocular blindness in children, 1945 to 1984.. Am J Dis Child.

[2] Hejtmancik JF, Smaoui N (2003). Molecular genetics of cataract.. Dev Ophthalmol.

[3] Hejtmancik JF (2008). Congenital cataracts and their molecular genetics.. Semin Cell Dev Biol.

[4] François J (1982). Genetics of cataract.. Ophthalmologica.

[5] Haargaard B, Wohlfahrt J, Fledelius HC, Rosenberg T, Melbye M (2004). A nationwide Danish study of 1027 cases of congenital/infantile cataracts: etiological and clinical classifications.. Ophthalmology.

[6] Chen J, Ma Z, Jiao X, Fariss R, Kantorow WL, Kantorow M, Pras E, Frydman M, Pras E, Riazuddin S, Riazuddin SA, Hejtmancik JF (2011). Mutations in FYCO1 Cause Autosomal-Recessive Congenital Cataracts.. Am J Hum Genet.

[7] Shiels A, Bennett TM, Hejtmancik JF (2010). Cat-Map: putting cataract on the map.. Mol Vis.

[8] Armitage MM, Kivlin JD, Ferrell RE (1995). A progressive early onset cataract gene maps to human chromosome 17q24.. Nat Genet.

[9] Kramer P, Yount J, Mitchell T, LaMorticella D, Carrero-Valenzuela R, Lovrien E, Maumenee I, Litt M (1996). A second gene for cerulean cataracts maps to the beta crystallin region on chromosome 22.. Genomics.

[10] Nandrot E, Slingsby C, Basak A, Cherif-Chefchaouni M, Benazzouz B, Hajaji Y, Boutayeb S, Gribouval O, Arbogast L, Berraho A, Abitbol M, Hilal L (2003). Gamma-D crystallin gene (CRYGD) mutation causes autosomal dominant congenital cerulean cataracts.. J Med Genet.

[11] Vanita V, Singh D, Robinson PN, Sperling K, Singh JR (2006). A novel mutation in the DNA-binding domain of MAF at 16q23.1 associated with autosomal dominant “cerulean cataract” in an Indian family.. Am J Med Genet A.

[12] Litt M, Carrero-Valenzuela R, LaMorticella DM, Schultz DW, Mitchell TN, Kramer P, Maumenee IH (1997). Autosomal dominant cerulean cataract is associated with a chain termination mutation in the human beta-crystallin gene CRYBB2.. Hum Mol Genet.

[13] Wang Q, Wang P, Li S, Xiao X, Jia X, Guo X, Kong QP, Yao YG, Zhang Q (2010). Mitochondrial DNA haplogroup distribution in Chaoshanese with and without myopia.. Mol Vis.

[14] Hansen L, Mikkelsen A, Nurnberg P, Nurnberg G, Anjum I, Eiberg H, Rosenberg T (2009). Comprehensive mutational screening in a cohort of Danish families with hereditary congenital cataract.. Invest Ophthalmol Vis Sci.

[15] Zhang Q, Zulfiqar F, Xiao X, Amer Riazuddin S, Ayyagari R, Sabar F, Caruso R, Sieving PA, Riazuddin S, Fielding Hejtmancik J (2005). Severe autosomal recessive retinitis pigmentosa maps to chromosome 1p13.3-p21.2 between D1S2896 and D1S457 but outside ABCA4.. Hum Genet.

[16] Lathrop GM, Lalouel JM (1984). Easy calculations of lod scores and genetic risks on small computers.. Am J Hum Genet.

[17] Schäffer AA, Gupta SK, Shriram K, Cottingham RW (1994). Avoiding recomputation in linkage analysis.. Hum Hered.

[18] Lander E, Kruglyak L (1995). Genetic dissection of complex traits: guidelines for interpreting and reporting linkage results.. Nat Genet.

[19] Terwilliger JD, Ott J. Handbook of Human Genetic Linkage. John Hopkins University Press: Baltimore; 1994.

[20] Jiang J, Jin C, Wang W, Tang X, Shentu X, Wu R, Wang Y, Xia K, Yao K (2009). Identification of a novel splice-site mutation in MIP in a Chinese congenital cataract family.. Mol Vis.

[21] Gu F, Zhai H, Li D, Zhao L, Li C, Huang S, Ma X (2007). A novel mutation in major intrinsic protein of the lens gene (MIP) underlies autosomal dominant cataract in a Chinese family.. Mol Vis.

[22] Wang W, Jiang J, Zhu Y, Li J, Jin C, Shentu X, Yao K (2010). A novel mutation in the major intrinsic protein (MIP) associated with autosomal dominant congenital cataracts in a Chinese family.. Mol Vis.

[23] Berry V, Francis P, Kaushal S, Moore A, Bhattacharya S (2000). Missense mutations in MIP underlie autosomal dominant 'polymorphic' and lamellar cataracts linked to 12q.. Nat Genet.

[24] Yang G, Zhang G, Wu Q, Zhao J (2011). A novel mutation in the MIP gene is associated with autosomal dominant congenital nuclear cataract in a Chinese family.. Mol Vis.

[25] Wang KJ, Li SS, Yun B, Ma WX, Jiang TG, Zhu SQ (2011). A novel mutation in MIP associated with congenital nuclear cataract in a Chinese family.. Mol Vis.

[26] Lin H, Hejtmancik JF, Qi Y (2007). A substitution of arginine to lysine at the COOH-terminus of MIP caused a different binocular phenotype in a congenital cataract family.. Mol Vis.

[27] Geyer DD, Spence MA, Johannes M, Flodman P, Clancy KP, Berry R, Sparkes RS, Jonsen MD, Isenberg SJ, Bateman JB (2006). Novel single-base deletional mutation in major intrinsic protein (MIP) in autosomal dominant cataract.. Am J Ophthalmol.

[28] Meyer E, Rahman F, Owens J, Pasha S, Morgan NV, Trembath RC, Stone EM, Moore AT, Maher ER (2009). Initiation codon mutation in betaB1-crystallin (CRYBB1) associated with autosomal recessive nuclear pulverulent cataract.. Mol Vis.

[29] Kolosha V, Anoia E, de Cespedes C, Gitzelmann R, Shih L, Casco T, Saborio M, Trejos R, Buist N, Tedesco T, Skach W, Mitelmann O, Ledee D, Huang K, Stambolian D (2000). Novel mutations in 13 probands with galactokinase deficiency.. Hum Mutat.

[30] Francis P, Chung JJ, Yasui M, Berry V, Moore A, Wyatt MK, Wistow G, Bhattacharya SS, Agre P (2000). Functional impairment of lens aquaporin in two families with dominantly inherited cataracts.. Hum Mol Genet.

